# A giant sporadic intra-abdominal desmoid tumor resection: a rare case report

**DOI:** 10.1016/j.ijscr.2025.111423

**Published:** 2025-05-09

**Authors:** Mohammad Al-Jawad, Abdoul Majid Sires, Aghyad Kurda Danial, Khadija Al-Haj Ali, Abdo M. Zain

**Affiliations:** University of Aleppo, Faculty of Medicine, Aleppo, Syria

**Keywords:** Desmoid tumors, Mesenchymal tumors, Immunohistochemistry, Case report

## Abstract

**Introduction:**

Desmoid tumors (DT) are rare fibroblastic proliferations with unpredictable clinical behavior, occurring at a rate of 2 to 4 cases per million annually, predominantly in females. Prognosis is variable, with spontaneous regressions in 20 % to 30 % of cases, and shorter progression-free survival is associated with younger age, larger tumor size, and extra-abdominal location.

**Case presentation:**

A 30-year-old female with chronic constipation and abdominal pain was diagnosed with a giant desmoid tumor originating from the mesentery, requiring surgical resection of the tumor, the right colon, and part of the ileum. Postoperatively, she recovered well and was referred to an oncologist for ongoing monitoring due to the tumor's potential for recurrence.

**Discussion:**

Desmoid tumors, or aggressive fibromatosis, are rare benign mesenchymal tumors characterized by local invasiveness, primarily affecting individuals aged 15 to 60, with a slight female predominance. Diagnosis is confirmed through histological evaluation and immunohistochemistry. Treatment typically involves local control through surgery and radiation, but there is a significant risk of recurrence, influenced by tumor size, location, and patient age.

**Conclusion:**

This case highlights the diagnostic challenges of desmoid tumors in the mesentery, demonstrated by a 30-year-old female patient with chronic abdominal pain. The tumor, one of the largest documented, required multidisciplinary management and careful surgical planning due to its location near major intestinal vessels, emphasizing the need for prompt action and specific clinical considerations.

## Highlights

• A 30-year-old female with chronic abdominal pain was diagnosed with a giant mesenteric desmoid tumor.
• Surgical resection was required due to its size and location.
• This case underscores the diagnostic challenges and recurrence risks associated with desmoid tumors.

## 1 Introduction

Desmoid tumor (DT) is a rare, monoclonal fibroblastic proliferation known for its variable and often unpredictable clinical behavior. In the International Classification of Diseases (ICD), it is categorized as D48.1 [1].

The incidence of desmoid tumor (DT) is low, with approximately 2 to 4 new cases diagnosed per million individuals each year. Based on their etiology, DT is classified into two main categories: sporadic DT and familial adenomatous polyposis (FAP)-associated DT. Sporadic DT accounts for 85 to 90 % of total diagnoses, and within this group, there is a notable female predominance, with a male-to-female ratio of about 0.5 [2].

The prognosis for patients with desmoid tumor (DT) is notoriously unpredictable. Tumors may exhibit an erratic disease course, with spontaneous regressions occurring in 20 % to 30 % of patients monitored over 2 to 3 years. Typically, an initial growth phase is followed by a period of stabilization. Factors significantly linked to shorter progression-free survival (PFS) include being younger than 37 years, having a tumor size greater than 7 cm, and the tumor's extra-abdominal location [3].

It is important to highlight that in our case, we present a 30-year-old female with giant intra-abdominal desmoid tumor and we highlight the challenges encountered in managing this unique case as per the SCARE checklist [4].

## 2 Case presentation

A 30-year-old female with a past medical history of psoriasis, which was effectively treated with topical cortisone over the past four months, presented with chronic constipation. Over the past two months, she experienced worsening right iliac fossa pain and urinary urgency, which progressed to dyspnea. Notably, she reported no hematochezia and maintained a regular menstrual cycle.

The urinary urgency reported by the patient may have resulted from the mass effect of the large abdominal tumor on the bladder or surrounding structures, contributing to increased pressure and irritation.

Upon clinical examination, a large, hard, mobile abdominal mass was palpated in the right lower quadrant. The mass was non-tender upon palpation. To further evaluate her condition, she underwent imaging studies, including an echography and multi-slice computed tomography (MSCT) scan.

**Fig. 1:**
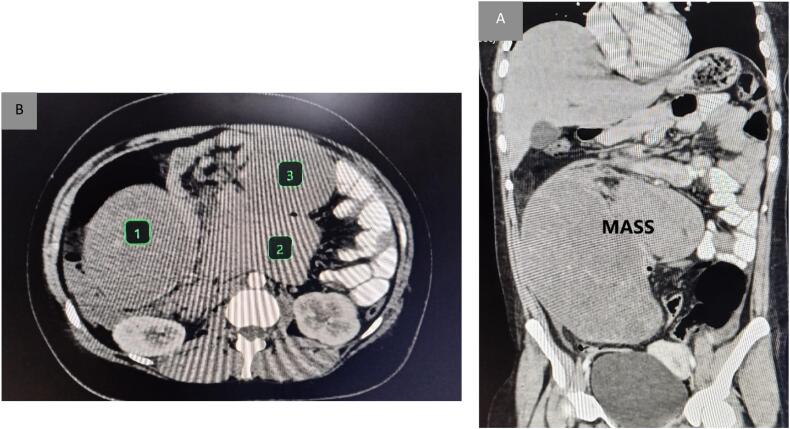
(a) Axial image from contrast-enhanced CT reveals three large connected mesenteric masses (stars) which are intimately associated with mesenteric vessels. (b) Coronal image from contrast-enhanced CT shows mildly enhancing low-attenuation mass in the abdomen (star).

Imaging studies reveal a substantial heterogeneous abdominal mass measuring 28 × 15 cm, originating from the mesentery of the small intestine. The mass is adherent to the superior mesenteric arteries; however, there is no evidence of invasion into adjacent structures. MSCT findings indicate that the mass extends from the inferior border of the pancreas to the uterus, displaying axial deviation without any apparent infiltration into the vascular structures. Importantly, the mass remains completely isolated from the uterus and bladder, with no signs of metastatic spread to surrounding organs, including the liver, spleen, and kidneys (Fig. 1a, b). These findings suggest a significant mass effect on the vascular structures while demonstrating a lack of invasion or infiltration into neighboring organs, potentially indicating a more favorable clinical prognosis.

Given the size and characteristics of the mass, surgical intervention was deemed necessary.

General anesthesia was administered for the surgical procedure to ensure the patient was unconscious and pain-free during the operation, facilitating a smoother surgical process.

**Fig. 2:**
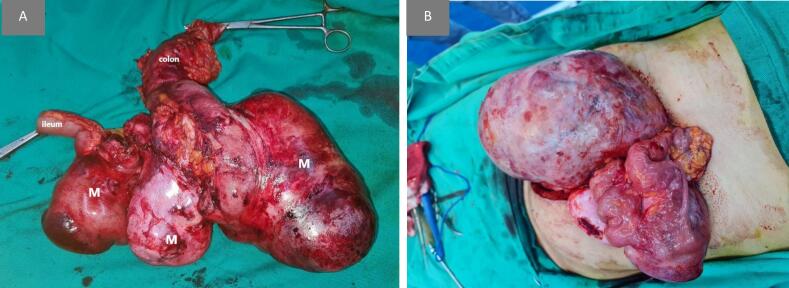
(a) Macroscopically, three attached hard elastic nodules measuring 14 × 25 cm, 12 × 8 cm and 14 × 8 cm lie mainly in the mesentery of small intestine; measuring in total 28 × 15 cm. (b) The mass during laparotomy pre to excision.

A midline incision was performed, during which extensive adhesions were encountered. The adhesiolysis proved challenging, particularly in preserving the superior mesenteric artery and its branches. Ultimately, en bloc resection of the tumor mass, the ascending colon, and approximately 50 cm of ileum was required (Fig. 2). An end-to-side ileocolic anastomosis was subsequently performed.

**Fig. 3:**
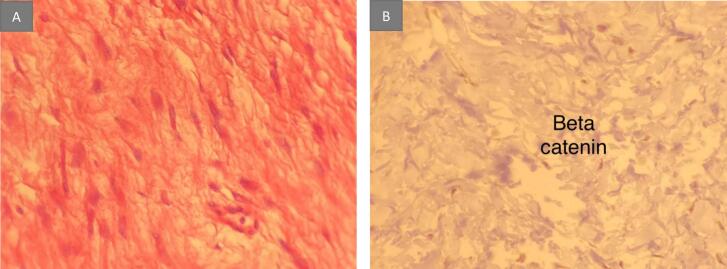
Pathologic features of the desmoid tumor. (a) Histologically, proliferation of spindle-shaped cells with collagenous stroma is seen (hematoxylin and eosin staining; high-power field). (b) Immunohistological examination reveals that the cell nuclei are positive for beta-catenin.

Histopathological analysis of the resected specimen revealed hypocellular spindle cells with prominent stromal hyalinization. Immunohistochemical staining confirmed the diagnosis of a desmoid tumor, characterized by positive beta-catenin expression and negative results for CD117 and S100 (Fig. 3a, b).

Postoperatively, the patient had an uneventful recovery and was discharged after 5 days of hospitalization. There were no complications such as ileus, infection, or anastomotic leakage. Following her discharge, she was referred to an oncologist for ongoing management and monitoring for potential recurrence, emphasizing the need for careful follow-up due to the nature of desmoid tumors.

The recommended follow-up protocol includes MRI scans every 6 to 12 months to monitor for any signs of recurrence, along with regular clinical evaluations to assess the patient's condition and address any concerns promptly.

## 3 Discussion

A desmoid tumor, commonly known as aggressive fibromatosis, is characterized by its local invasiveness and primarily originates from muscle, fascia, and various connective tissues. According to the World Health Organization (WHO), these rare benign mesenchymal tumors exhibit local aggressiveness and account for approximately 0.03 % of all neoplasms and less than 3 % of all soft tissue tumors. They are typically found outside the abdomen, such as in the abdominal wall, limbs, and girdles. Less commonly, they occur within the abdomen, often located in the mesentery [2,5,6].

In our case, the mass in the patient was located in the mesentery.

Individuals aged 15 to 60 are the most frequently affected by desmoid tumors, which are rare in both younger individuals and older adults. These tumors occur slightly more often in females than in males, and there is no significant predisposition based on race or ethnicity. Due to their rarity, desmoid tumors may be misdiagnosed in approximately 30 % to 40 % of cases, resulting in inappropriate or delayed treatment [7,8].

Patients with intra-abdominal desmoid tumors may experience symptoms such as weight loss, cachexia, and general malaise. Both sporadic and familial adenomatous polyposis (FAP)-associated desmoid tumors can significantly impact the quality of life (QoL) of patients, negatively affecting physical, social, cognitive, and emotional aspects [3].

In our case, a 30-year-old female presented to the hospital with pain in the right iliac fossa, accompanied by respiratory complaints for two months, but without nausea or vomiting. This presentation facilitated a rapid diagnosis and prevented delays in treatment.

The precise nature of her condition—whether it is an isolated occurrence or associated with FAP—remains unclear. However, the results from the clinical history, and pathology findings strongly suggest that it is indeed an isolated case. The patient has no family history of FAP and does not exhibit symptoms typically associated with this condition. Additionally, the absence of any polyps in the resected sections of the colon further supports this diagnosis.

The diagnosis of a desmoid tumor is confirmed through histological evaluation of a biopsy specimen. Typically, an incisional biopsy offers a larger tissue sample compared to a core needle biopsy, which aids in distinguishing between benign and malignant conditions. However, experienced pathologists may find that a core biopsy can provide adequate tissue for an accurate diagnosis [8].

Immunohistochemistry can assist in the histologic diagnosis of desmoid tumors, as spindle cells typically show positive staining for vimentin, smooth muscle actin, and nuclear beta-catenin, while generally being negative for desmin, cytokeratins, and S-100. Notably, nuclear beta-catenin immunoreactivity reinforces the diagnosis, with positive staining observed in 80 % of sporadic desmoids and 67 % of FAP-associated desmoids in a large study [8,9].

However, this finding is not definitive, as other conditions such as superficial fibromatoses, low-grade myofibroblastic sarcomas, and solitary fibrous tumors can also exhibit nuclear beta-catenin staining. Additionally, a negative result for beta-catenin does not rule out a diagnosis of fibromatosis. Next-generation sequencing has been shown to be highly sensitive in detecting CTNNB1 mutations in desmoid-type fibromatoses [9,10].

In our case, all necessary investigations, including echography and multi-slice computed tomography (MSCT), were conducted, and their results were previously outlined. After the surgical procedure, a tissue sample was sent for histopathological examination, confirming the diagnosis of a desmoid tumor. The analysis revealed hypocellular spindle cells with prominent stromal hyalinization, characteristic of desmoid tumors. Immunohistochemical staining confirmed the diagnosis through positive beta-catenin expression, consistent with literature findings. Negative results for CD117 and S100 further supported the diagnosis, as these markers are typically absent in desmoid tumors.

Due to their inability to metastasize, local control through surgery and radiation has traditionally been the primary treatment for desmoid tumors. However, there is a notable risk of local recurrence, even following complete surgical removal. A multidisciplinary management approach is becoming more common, especially for patients with intra-abdominal desmoids. The anatomical location of the desmoid tumor is a key factor influencing treatment strategies. Additionally, the most significant predictors of recurrence after surgery are tumor size, location, and the age of the patient [2,7].

In our case, we faced significant surgical challenges, particularly with adhesiolysis and the preservation of the superior mesenteric artery, highlighting the complexity of the procedure. The decision to resect the ascending colon and a 50 cm of the ileum was crucial for achieving complete tumor resection and ensuring gastrointestinal continuity. Postoperatively, follow-up with an oncologist is essential due to the potential recurrence of desmoid tumors, emphasizing the need for long-term management. Ultimately, the patient's uneventful recovery reflects the success of our surgical approach and postoperative care.

It is important to note that the tumor in our case is considered the largest documented desmoid tumors in the medical literature. Additionally, the timely treatment provided to the patient has prevented the progression of the condition and the deterioration of her health.

## 4 Conclusion

This case illustrates the challenges of diagnosing desmoid tumors, particularly those located in the mesentery. Our 30-year-old female patient was diagnosed during investigations prompted by her chronic abdominal pain. Multidisciplinary management and thorough histological evaluation led to effective treatment. Notably, the tumor is one of the largest documented desmoid tumors, highlighting the importance of awareness and prompt action.

The resection of such a large tumor presents significant challenges due to its location in the mesentery, which contains major vessels supplying the intestine. Preserving these vessels during surgery is crucial, as damage could lead to severe complications. This emphasizes the need for careful planning and specific clinical considerations to ensure a successful procedure while minimizing risks to the patient's health.

## Author contribution

The work's conception and design: Mohammad Al-Jawad - ABDO M.ZAIN.

paper writing, and article revision: Mohammad Al-Jawad - Abdoul Majid Sires - Khadija Al-Haj Ali.

Final revision and approval: Mohammad Al-Jawad - Aghyad Kurda Danial - ABDO M.ZAIN.

## Consent

Written informed consent was obtained from the patient for publication and any accompanying images. A copy of the written consent is available for review by the Editor-in-Chief of this journal on request.

## Ethical approval

This case report does not require ethical approval in our institution (University of Aleppo) as it involves a single patient case that is anonymized and does not include any identifiable personal information. The patient provided informed consent for the publication of this report.

## Guarantor

ABDO M. ZAIN.

## Research registration number

Our research study does not involve human subjects.

## Provenance and peer review

Not commissioned, externally peer-reviewed.

## Funding

This research did not receive any funding from any external sources. All activities related to this research were conducted without financial support.

## Conflict of interest statement

The authors declare that they have no competing interests.
